# Does Coenzyme Q10 Plus Selenium Supplementation Ameliorate Clinical Outcomes by Modulating Oxidative Stress and Inflammation in Individuals with Myalgic Encephalomyelitis/Chronic Fatigue Syndrome?

**DOI:** 10.1089/ars.2022.0018

**Published:** 2022-04-19

**Authors:** Jesús Castro-Marrero, Joan Carles Domingo, Begoña Cordobilla, Roser Ferrer, Marina Giralt, Ramon Sanmartín-Sentañes, Jose Alegre-Martín

**Affiliations:** ^1^ ME/CFS Research Unit, Division of Rheumatology, Vall d'Hebron Research Institute, Universitat Autònoma de Barcelona, Barcelona, Spain.; ^2^ Department of Biochemistry and Molecular Biomedicine, Faculty of Biology, University of Barcelona, Barcelona, Spain.; ^3^ Department of Clinical Biochemistry, Vall d'Hebron University Hospital, Universitat Autònoma de Barcelona, Barcelona, Spain.; ^4^ ME/CFS Clinical Unit, Division of Rheumatology, Vall d'Hebron University Hospital, Universitat Autònoma de Barcelona, Barcelona, Spain.

**Keywords:** biomarkers, cardiovascular health, coenzyme Q10, chronic fatigue syndrome, inflammation, myalgic encephalomyelitis, redox status, selenium

## Abstract

Myalgic encephalomyelitis/chronic fatigue syndrome (ME/CFS) is a neuroinflammatory, multifaceted chronic disorder of unknown cause. Accumulating data indicate a link between a redox imbalance, mitochondrial dysfunction, and inflammation status in ME/CFS. Coenzyme Q10 (CoQ10) and selenium as effective antioxidant and anti-inflammatory agents have shown potential clinical implications in chronic diseases; however, their therapeutic benefits in ME/CFS remain elusive. This open-label exploratory study aimed to evaluate the effectiveness of combined CoQ10 plus selenium supplementation on clinical features and circulating biomarkers in ME/CFS. Twenty-seven ME/CFS patients received an oral combination of 400 mg of CoQ10 and 200 μg of selenium daily for 8 weeks. The primary endpoint was patient-reported changes in outcome measures from baseline to 8 weeks' postintervention. Secondary endpoint included changes in circulating biomarkers from baseline to each participant. After an 8-week intervention, a significant improvement was found for overall fatigue severity (*p* = 0.021) and global quality of life (*p* = 0.002), while there was no significant effect on the sleep disturbances (*p* = 0.480) among participants. After 8 week's intervention, there was significantly increased total antioxidant capacity, and there were reduced lipoperoxide levels from the participants (*p* < 0.0001 for both). Circulating cytokine levels decreased significantly (*p* < 0.01 for all), but with no significant changes in the C-reactive protein, FGF21, and NT-proBNP biomarkers after supplementation. Based on these findings, we hypothesized that long-term supplementation of combined CoQ10 and selenium may indicate a potentially beneficial synergistic effect in ME/CFS. *Antioxid. Redox Signal.* 36, 729–739.

**Figure:**
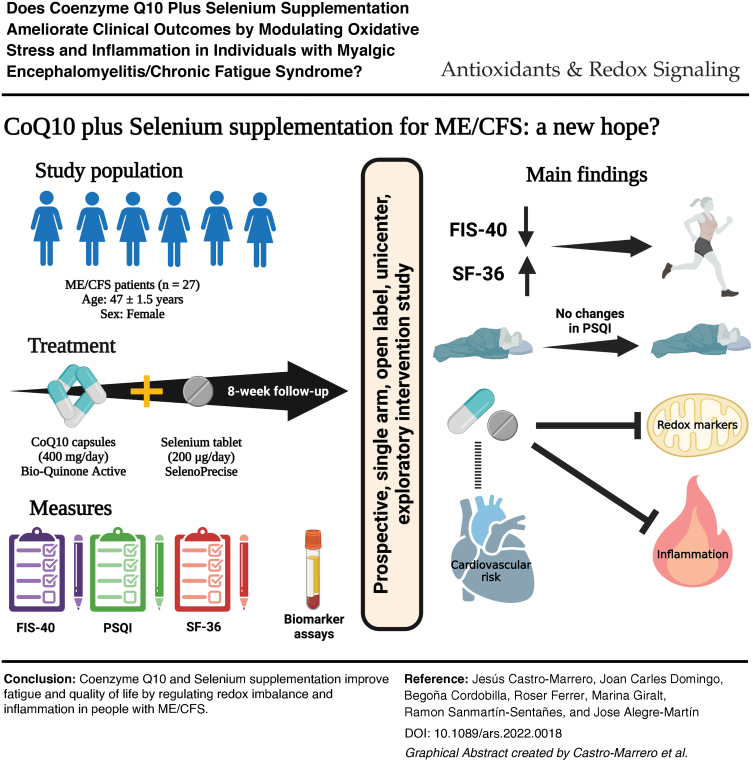
*Color images are available online.*

## Introduction

Myalgic encephalomyelitis, also commonly referred to as chronic fatigue syndrome (ME/CFS), is a serious, debilitating, multifaceted chronic disorder of unknown etiology recognized by the WHO and U.S. Centers for Disease Control and Prevention (CDC) as a neurological illness (7). ME/CFS is characterized by unexplained overwhelming fatigue, postexertional malaise considered as a hallmark feature, unrefreshing sleep, neurocognitive impairments, gastrointestinal complaints, and orthostatic intolerance often worsening after minimal physical and mental exertion. These postexertional symptoms can persist for hours, days, or even weeks, and are not substantially alleviated by rest or sleep (14).

To date, there is no objective diagnostic test, the pathomechanism remains unclear, and there are no targeted effective drugs available for ME/CFS. Therefore, the diagnosis is presently based on clinical presentation of symptoms according to the case criteria when other potential fatiguing-associated conditions are excluded (4, 9).

ME/CFS is a prevalent condition affecting ∼50 million people globally and currently causing a huge burden on people of working age. The CDC and U.S. National Academy of Medicine estimate that 836,000 to 2.5 million people have ME/CFS in the United States alone. In Europe, the prevalence has been documented with >3 million people suffering from ME/CFS. In the coming years, the largest increase in ME/CFS prevalence from COVID-19 is expected to affect 152 million by 2050, a challenge still unresolved by global health system (11).

In the last few years, there has been a growing interest in the potential role that mitochondria may play in the pathogenesis of ME/CFS. Possible common pathomechanisms of mitochondrial disruption (including oxidative stress, redox imbalance, and impaired ATP production) and low-grade systemic inflammation in ME/CFS have been extensively reviewed in the literature (15). The use of different supplements has been widely documented in ME/CFS either alone or in combination as cocktail, including coenzyme Q10 (CoQ10), l-carnitine, NADH, d-ribose, vitamins, magnesium, melatonin, zinc, folic acid, essential fatty acids, and *N*-acetyl-l-carnitine as part of their concomitant treatment regime in the attempt to ameliorate the patients' symptoms (12).

Previous studies by our group (5, 6) have demonstrated a beneficial effect of CoQ10 supplementation on clinical symptoms and mitochondrial function in ME/CFS; however, no randomized-controlled trials (RCTs) have been conducted using CoQ10 combined with selenium in ME/CFS patients. Most adults in Spain have circulating selenium levels below the optimum range (10). Selenium is a key player in regulating immunity health and inflammation in several chronic conditions, including ME/CFS. In addition, selenium is an essential micronutrient required of ∼25 different selenoproteins in humans (3).

The rationale to combine selenium with CoQ10 is based on the need for the selenium-dependent thioredoxin reductase enzyme to reduce CoQ10. A deficiency of selenium could therefore restrict the cells' ability to obtain the optimal concentrations of ubiquinol. We hypothesized that combined CoQ10 plus selenium supplementation could mitigate clinical symptoms by modulating oxidative stress and systemic inflammation in ME/CFS.

**Innovation**

Redox imbalance and chronic low-grade inflammation have been implicated in myalgic encephalomyelitis/chronic fatigue syndrome (ME/CFS). No drug therapies exist in ME/CFS, so antioxidant and anti-inflammatory supplements have been focused on the symptom management in the condition. This study is the first exploratory open-label trial of coenzyme Q10 (CoQ10) plus selenium supplementation in ME/CFS patients. Oral CoQ10 plus selenium administration ameliorates perceived fatigue severity and quality of life by modulating oxidative stress and inflammatory status in ME/CFS. Based on these observations, we hypothesize that this study may indicate a beneficial synergistic effect of combined CoQ10 plus selenium administration in ME/CFS. Further investigation should be done to elucidate novel antioxidant supplementation-based therapeutic designs to ameliorate clinical features in ME/CFS.

Given the aforementioned evidence, future well-designed interventions using nutritional supplements that target the mitochondria are needed to elucidate the potential therapeutic effects of these approaches in ME/CFS (16). The present trial aimed to investigate the efficacy of combined oral CoQ10 and selenium supplementation on clinical outcomes (fatigue, sleep disturbances, and quality of life) and circulating biomarkers associated with redox status, inflammation, and cardiovascular health in ME/CFS.

## Results and Discussion

### Demographic and clinical characteristics of the study population

**FIG. 1.**
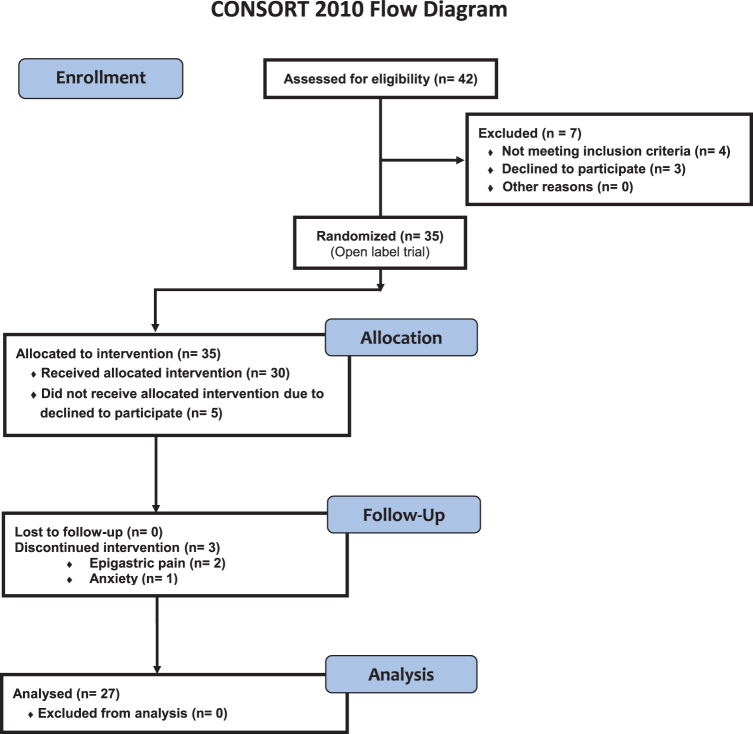
**Overview of clinical trial protocol illustrating the experimental design of the participants.** Oral 400 mg CoQ10 plus 200 μg selenium per day was supplemented to each participant over 8 weeks of intervention. CoQ10, coenzyme Q10. Color images are available online.

Relevant demographic, clinical characteristics and routine blood tests of the study participants are summarized in Table 1. Forty-two adult ME/CFS patients with mild/moderate symptoms who met the 1994 CDC/Fukuda definition were potentially eligible for the study. From this original sample, seven (16.7%) were not included in the study, because they did not meet the inclusion criteria (*n* = 4), or because they declined to participate (*n* = 3) (Fig. 1). The mean age of the participants was 47.3 ± 1.5 years and all were female.

**Table 1.** Demographic and Clinical Characteristics and Laboratory Parameters from the Enrolled Participants

Variables	Baseline	Post-treatment	p^a^
Age, years	47.3 ± 1.5	47.3 ± 1.5	1.000
BMI, kg/m^2b^	23.9 ± 0.5	22.8 ± 1.2	0.942
Systolic BP, mmHg	121.8 ± 2.5	119.4 ± 1.7	0.750
Diastolic BP, mmHg	82.3 ± 1.6	80.1 ± 2.2	0.831
HR, bpm	70.1 ± 1.8	72.5 ± 1.4	0.893
Routine blood test panel			
RBC, × 10^12^/L	4.6 ± 0.07	4.5 ± 0.08	**0.036**
Hemoglobin, g/dL	13.5 ± 0.25	13.4 ± 0.26	0.224
Hematocrit, %	41.2 ± 0.65	40.7 ± 0.68	0.059
Neutrophils, × 10^9^/L	3.7 ± 0.33	3.9 ± 0.39	0.842
Lymphocytes, × 10^9^/L	2.0 ± 0.13	2.2 ± 0.13	**0.011**
Monocytes, × 10^9^/L	0.45 ± 0.04	0.51 ± 0.04	**0.003**
Eosinophils, × 10^9^/L	0.14 ± 0.02	0.15 ± 0.02	0.754
Basophils, × 10^9^/L	0.03 ± 0.009	0.02 ± 0.008	0.375
Platelets, × 10^9^/L	294.4 ± 29.3	279.2 ± 19.4	0.296
Glucose, mg/dL	93.7 ± 2.88	98.2 ± 2.94	**0.006**
Urea, mg/dL	32.4 ± 1.501	31.9 ± 1.384	0.766
Creatinine, mg/dL	0.67 ± 0.02	0.68 ± 0.02	0.297
Urate, mg/dL	4.45 ± 0.253	4.54 ± 0.206	0.527
Total bilirubin, mg/dL	0.56 ± 0.052	0.51 ± 0.044	0.052
Indirect bilirubin, mg/dL	0.21 ± 0.010	0.02 ± 0.009	0.113
Cholesterol, mg/dL	232.0 ± 7.64	219.4 ± 5.87	**0.011**
Triglycerides, mg/dL	133.3 ± 10.93	138.3 ± 13.53	0.883
LDL, mg/dL	142 ± 6.62	130 ± 4.97	**0.012**
HDL, mg/dL	62.1 ± 2.36	61.5 ± 2.53	0.609
Total protein, g/dL	7.1 ± 0.062	7.0 ± 0.068	**0.048**
Albumin, g/dL	4.38 ± 0.046	4.31 ± 0.042	0.106
AST, UI/L	21.0 ± 0.90	20.6 ± 0.81	0.311
ALT, UI/L	19.5 ± 1.78	20.6 ± 1.79	0.929
ALP, UI/L	81.2 ± 4.61	80.3 ± 4.90	0.494
GGT, UI/L	27.9 ± 4.22	29.0 ± 3.49	0.068
TSH, mUI/L	1.751 ± 0.19	2.347 ± 0.32	**0.0045**
Free-T4, ng/dL	1.181 ± 0.03	1.076 ± 0.02	**<0.0001**
Measures			
FIS-40			
Global score (0–160)	137.7 ± 3.6	129.3 ± 4.5	**0.021**
Physical	35.7 ± 0.9	33.6 ± 1.2	**0.007**
Cognitive	34.9 ± 0.7	32.8 ± 1.0	**0.035**
Psychosocial	67.1 ± 2.1	62.8 ± 2.5	0.054
PSQI			
Global score (0–21)	15.9 ± 0.8	15.3 ± 0.8	0.480
Subjective sleep quality	2.3 ± 0.2	2.3 ± 0.2	0.945
Sleep latency	2.7 ± 0.1	2.7 ± 0.1	1.000
Sleep duration	1.8 ± 0.2	1.9 ± 0.2	0.531
Habitual sleep efficiency	2.1 ± 0.2	1.8 ± 0.2	0.285
Sleep disturbances	2.1 ± 0.1	2.1 ± 0.1	0.727
Sleeping medication	2.2 ± 0.2	2.1 ± 0.2	0.812
Daytime dysfunction	2.5 ± 0.1	2.2 ± 0.2	0.138
SF-36			
Global score (0–100)	22.7 ± 2.9	30.6 ± 3.0	**0.002**
Physical functioning	27.4 ± 3.7	30.7 ± 3.7	0.334
Physical role	0.9 ± 0.8	10.2 ± 4.6	0.063
Bodily pain	14.7 ± 3.2	22.0 ± 3.9	**0.015**
General health perception	22.3 ± 3.4	26.0 ± 3.4	0.151
Vitality	13.7 ± 3.1	15.9 ± 3.5	0.151
Social role functioning	32.4 ± 4.7	39.4 ± 4.4	0.143
Emotional role functioning	30.8 ± 8.5	51.9 ± 10.6	**0.023**
Mental health	39.8 ± 3.4	48.4 ± 3.5	**0.048**
Medications, *n* (%)^c^			
NSAIDs	9 (42.9)	6 (28.6)	0.68
Hypnotics	5 (23.8)	4 (27.0)	0.89
Sedatives/antidepressants	6 (28.6)	6 (28.6)	1.00
Antipsychotics	4 (19.0)	3 (14.3)	0.91
Opioids	11 (52.4)	9 (42.9)	0.88

Patient-reported outcome measures (overall and domain scores) taken before (baseline) and after coenzyme Q10 plus selenium supplementation (8-week post-treatment), as explained in the Notes section. Unless otherwise indicated, values are presented as mean ± SEM for parametric variables and as number of cases (percentages) for categorical variables who are taking drugs in the intervention.

^a^ Data were analyzed using the nonparametric Wilcoxon signed-rank test when appropriate. Bold values denote statistical significance at *p* < 0.05 (compared with baseline).

^b^ The BMI is the weight in kilograms divided by the square of the height in meters.

^c^ In addition, ∼70% of subjects are taking more than one medication as usual care treatment.

ALP, alkaline phosphatase; ALT, alanine aminotransferase; AST, aspartate aminotransferase; BMI, body mass index; BP, blood pressure; FIS-40, 40-item fatigue impact scale; GGT, gamma-glutamyl transferase; HDL, high-density lipoprotein; HR, heart rate; LDL, low-density lipoprotein; NSAIDs, nonsteroidal anti-inflammatory drugs; PSQI, Pittsburgh Sleep Quality Index; RBC, red blood cells; SEM, standard error of the mean; SF-36, short-form 36-item health survey; T4, thyroxine; TSH, thyroid-stimulating hormone.

As shown in Table 1, there were no significant differences in age, body mass index (BMI), blood pressure (BP), or heart rate at baseline and after 8 weeks of treatment. The biochemical markers in which we found statistically significant differences after the intervention (low-density lipoprotein [LDL], cholesterol, TSH, and free T4) are in agreement with previous results using CoQ10 and selenium supplementation.

As shown in Table 1, statistically significant differences were found in the scores for perceived overall fatigue assessed by 40-item fatigue impact scale (FIS-40) questionnaire (*p* = 0.021) over the course of the clinical study. Physical and cognitive fatigue perception improved significantly at the end of the intervention among participants (*p* = 0.007 and *p* = 0.035, respectively). The global short-form 36-item health survey (SF-36) score showed statistically significant improvements at week 8 of intervention (*p* = 0.002). The bodily pain, emotional role functioning, and mental health domains improved from baseline (*p* = 0.015, *p* = 0.023, and *p* = 0.048, respectively).

No statistically significant differences were found for sleep quality assessed using the Pittsburgh Sleep Quality Index (PSQI) questionnaire at the end of intervention in the participants. No statistically significant differences were observed in the standard medication therapy used among the participants.

### Effect of CoQ10 plus selenium supplementation on redox status

**FIG. 2.**
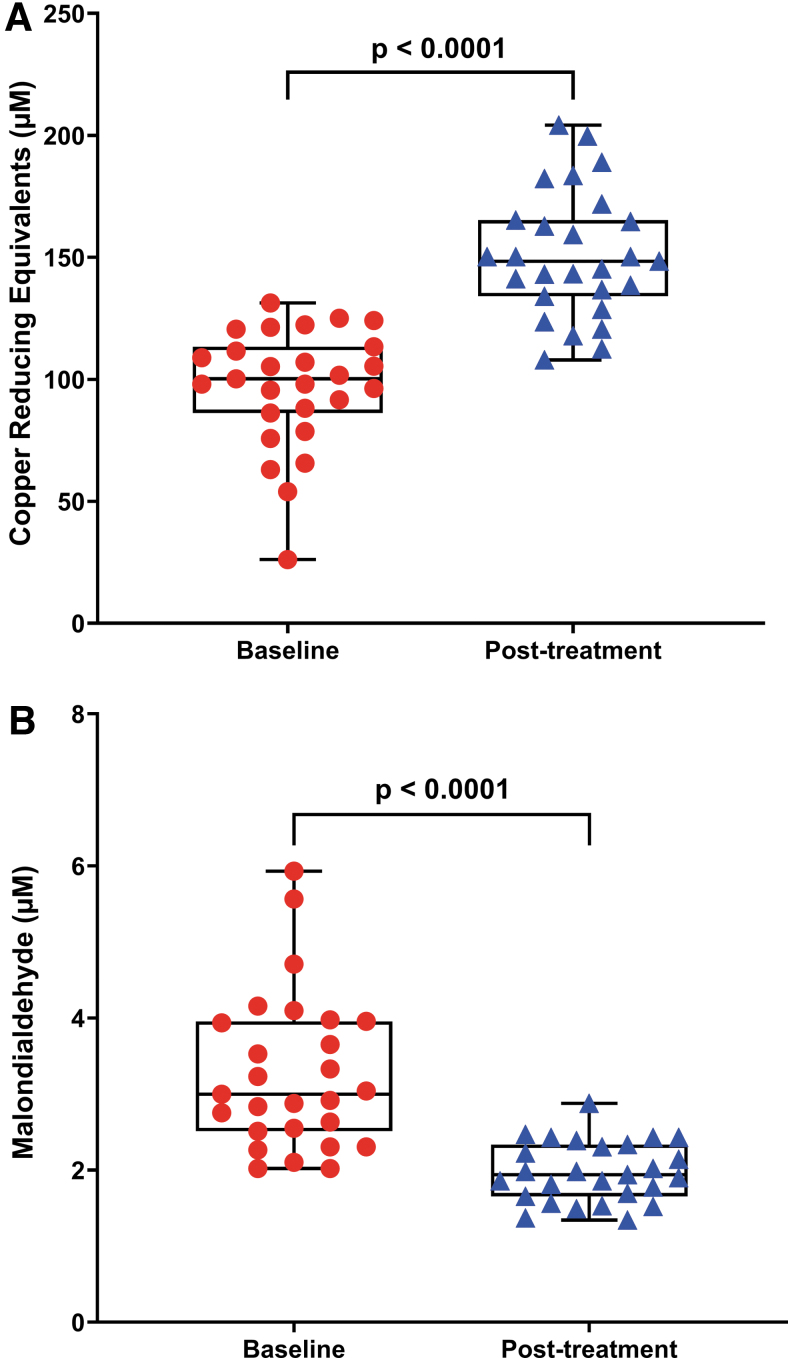
**Reduction of oxidative stress status after CoQ10 and selenium supplementation among participants.** Representative box plot diagram showing statistically significant differences of TAC (expressed as μ*M* of copper-reducing equivalents) and lipid peroxidation levels (assessed by TBARS assay) **(A, B)** after 8 weeks' intervention from baseline. Each *dot* represents a single participant (*n* = 27). Data are expressed as mean ± SEM of duplicates and are representative of two independent experiments. The *box* extends from the 25th to 75th percentiles, the *line* represents the mean, and the *whiskers* show the minimum and maximum. *p*-Values *versus* baseline were calculated using the Wilcoxon signed-rank test. SEM, standard error of the mean; TAC, total antioxidant capacity; TBARS, thiobarbituric acid-reacting substances. Color images are available online.

Figure 2 shows the levels of total antioxidant capacity (TAC) expressed as μ*M* copper-reducing equivalents (Fig. 2A) and lipid peroxidation assessed by malondialdehyde (MDA) levels (Fig. 2B) in plasma before and after the combined supplementation in the participants. There was a statistically significant increase in the TAC and a statistically significant decrease in the lipoperoxide content (*p* < 0.0001 for both) after CoQ10 plus selenium supplementation among participants. In accordance with a previous study, these findings stress the increased redox imbalance in ME/CFS, which could be modulated by CoQ10 plus selenium supplementation in individuals with ME/CFS (8).

### Effect of CoQ10 plus selenium supplementation on circulating inflammatory markers

**FIG. 3.**
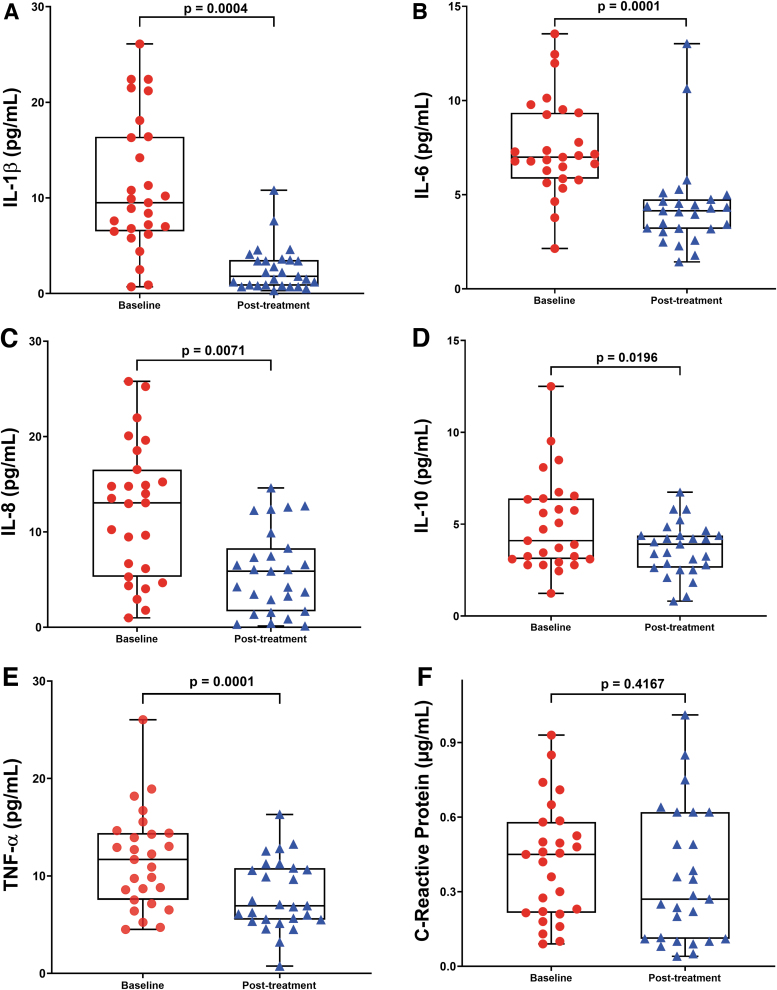
**Decreased inflammatory profile after CoQ10 plus selenium supplementation in the study participants. (A–E)** The circulating levels of inflammatory cytokines and C-reactive protein **(F)** at baseline and after 8-week CoQ10 plus selenium administration in participants as described in the Notes section. Each *dot* represents a single participant (*n* = 27). Values are shown as mean ± SEM of duplicates and are representative of two independent experiments. The *box* extends from the 25th to 75th percentiles, the *line* represents the mean, and the *whiskers* show the minimum and maximum. *p*-Values *versus* baseline were calculated using the Wilcoxon signed-rank test. Color images are available online.

Figure 3 shows the levels of inflammatory cytokines (IL-1β, IL-6, IL-8, IL-10, TNF-α) and C-reactive protein (CRP) in the study participants. As expected, the inflammatory cytokine levels showed statistically significant decreases (*p* < 0.01 for all) in the study participants at 8 weeks after supplementation (Fig. 3A–E). However, blood CRP levels did not show statistically significant differences between subjects participating in the study after 8 weeks' combined supplementation (*p* = 0.4167; Fig. 3F).

A recent meta-analysis study showed that CoQ10 supplementation can reduce significantly serum high-sensitivity C-reactive protein (hs-CRP) level only in cardiovascular disease patients with elevated baseline hs-CRP levels (>3 mg/L) or when the intervention duration was >12 weeks (2). Controlling for the potential confounders (age and BMI) did not affect the results of this study (data not shown).

### Effect of CoQ10 plus selenium supplementation on cardiovascular health

**FIG. 4.**
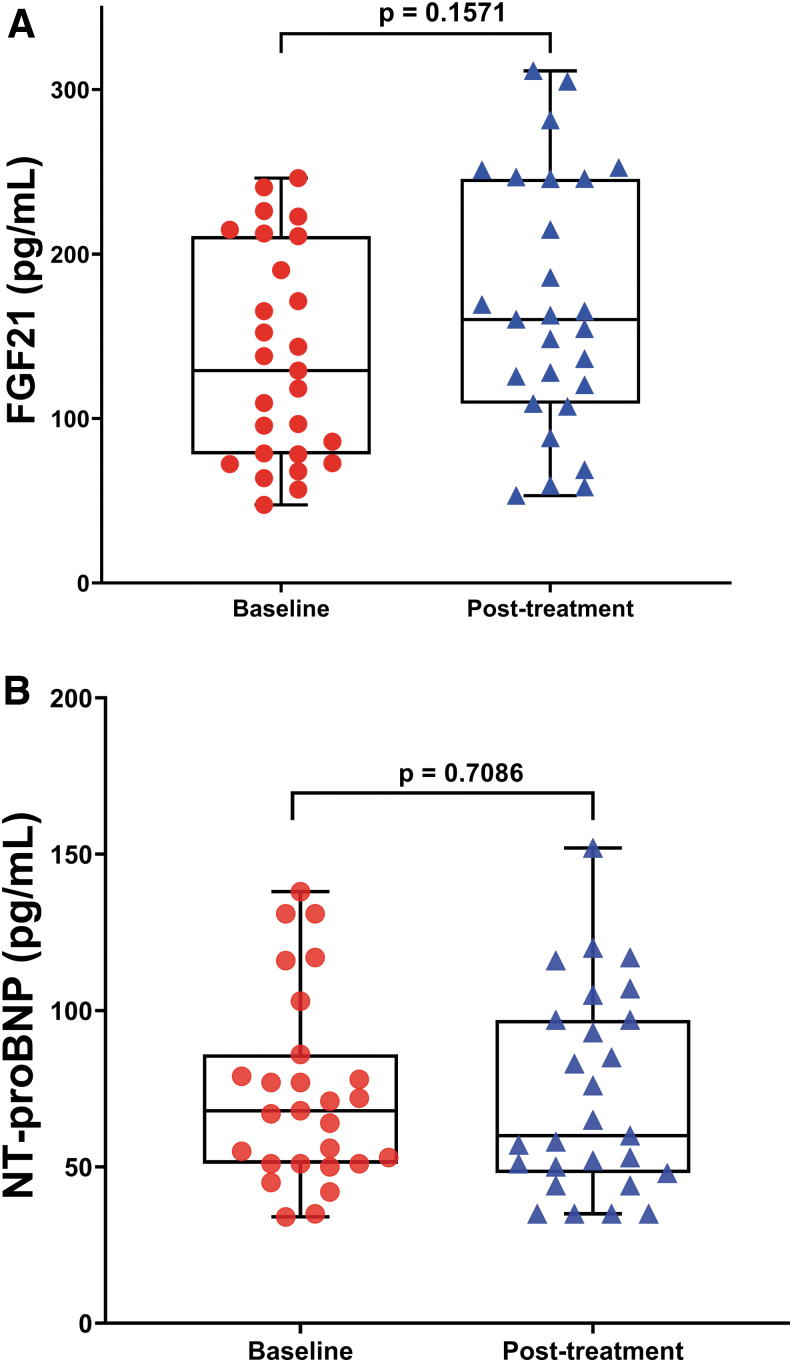
**Biomarkers of cardiovascular risk do not change during the intervention in the participants.** Representative box plots showing no statistically significant differences of circulating FGF21 and NT-proBNP levels **(A, B)** between baseline and 8 weeks of intervention. Each *dot* represents a single participant (*n* = 27). Values are expressed as mean ± SEM of duplicates and are representative of two independent experiments. The *box* extends from the 25th to 75th percentiles, the *line* represents the mean, and the *whiskers* show the minimum and maximum. *p*-Values *versus* baseline were calculated using the Wilcoxon signed-rank test. FGF21, fibroblast growth factor 21; NT-proBNP, N-terminal prohormone of brain natriuretic peptide. Color images are available online.

As shown in Figure 4, plasma levels of FGF21 and NT-proBNP were not significantly different after 8 weeks of CoQ10 plus selenium administration (*p* = 0.1571 and *p* = 0.7086, respectively) among the participants. A previous RCT study (2013 KiSel-10) was conducted in an elderly Swedish population to explore the effect of oral CoQ10 plus selenium administration on cardiovascular health by assessing circulating NT-proBNP levels at baseline and after 48 months' intervention. The authors concluded that long-term (4 years) CoQ10 plus selenium supplementation significantly reduced NT-proBNP levels and cardiovascular risk in those with increased baseline NT-proBNP levels, indicating that those who gain from the combined supplementation are patients with mild-to-moderate impaired cardiac function (1).

In the Q-Symbio study of the effect of CoQ10 alone (*i.e.,* no selenium supplementation) only as adjuvant therapy for chronic heart failure patients, the researchers observed no significant effect on NT-proBNP by the CoQ10 supplementation (2 years of three times 100 mg CoQ10 supplementation daily); however, there was a trend with a mean reduction of 384 pg/mL (20%) of NT-proBNP levels in the CoQ10 group and a proportional rise of 199 pg/mL (12%) of NT-proBNP in the placebo group (13). No reports exist on the role of FGF21 and cardiovascular function after supplementation of CoQ10 and selenium in ME/CFS patients.

Taken together, our short-term findings provide evidence for the first time that combined CoQ10 plus selenium supplementation ameliorates clinical symptoms by modulating oxidative stress and inflammation markers in ME/CFS, and may therefore be a promising novel supplementation-based therapeutic option in ME/CFS in long-term interventions.

### Limitations and strengths of the study

This study is strengthened by its prospective design, systematic data collection, and assessment of comprehensive circulating biomarkers involved in redox imbalance, systemic inflammation, and cardiovascular function in people with ME/CFS. Several limitations of this trial should be considered. First, this study included patients who met the 1994 CDC/Fukuda definition for ME/CFS. While this case criterion is the most widely utilized, it is considered too broad and represents a more heterogeneous set of ME/CFS patients with overlap with other comorbid illnesses such as depression (9).

Applying stricter ME/CFS case criteria including the 2003 CCC, 2011 ME-ICC, and/or 2015 NIH/IOM (SEID) may improve the overall sensitivity and specificity of study population selection, which is an important consideration for further interventions in ME/CFS. Second, the patient cohort was too small (*n* = 27) to draw any significant findings with respect to clinical outcomes. However, since the aim of this study was to examine whether CoQ10 plus selenium supplementation for 8 weeks was sufficient to improve clinical features and circulating biomarkers linked to redox status, systemic inflammation, and cardiovascular health in ME/CFS, we believe the sample size used here was adequate.

Nevertheless, we cannot rule out the existence of residual confounders due to the small sample size and comorbid health conditions in our ME/CFS cohort. Third, since the study design is a pilot, single-arm open-label trial in which an established placebo-controlled arm is lacking. Therefore, implementation of a placebo-controlled group is important to account for the placebo effect contribution to the observed results especially where subjective outcome measures are concerned. Overall improvement in study design in future studies is paramount, including the replication of open pilot studies as an RCT.

Fourth, this trial explored the effects of CoQ10 plus selenium supplementation in a short-term duration (8 weeks of treatment). Replication of these findings for longer durations to assess its long-term implications is needed for future interventions. These findings, and others, have informed the rationale for further clinical study and are strengths of this study. Since blood samples were only obtained before the intervention and at the end of the study, it was not possible to determine the temporality of adaptations that occurred while the CoQ10 plus selenium intervention was ongoing.

Our study recruited only Caucasian females to give sample homogeneity. Lack of inclusion of minority groups, however, will result in a sample that does not appropriately represent the population. Information (data) on diet restrictions was not recorded in this study. Due to possible food–drug–supplement and food–food interactions, consumption of certain foods may influence response to treatment; hence, a diet record (nutritional intake) is a necessary consideration for future mitochondrial-targeting nutraceutical interventions.

## Concluding Remarks on Future Directions

This study confirms a beneficial synergistic effect of CoQ10 plus selenium administration on clinical outcomes by decreasing oxidative stress and inflammatory biomarkers after 8 weeks of treatment in the study population. This study did not find any significant changes in the levels of FGF21 and NT-proBNP after 8 weeks of intervention among the participants; however, we found statistically significant decreased levels for LDL-cholesterol in blood, which suggests that these findings may be implicated in the reduction of cardiovascular risk factors secondary to illness. Moreover, this short-term trial using 400 mg of CoQ10 plus 200 μg of selenium administration per day was well tolerated from ME/CFS patients.

Further studies are needed to determine whether these early promising results utilizing CoQ10 with selenium supplementation prove to be beneficial in long-term randomized, placebo-controlled trials.

## Notes

### Ethics statement

The protocol study was approved by the local Ethics Committee on Biomedical Research at the Vall d'Hebron University Hospital in Barcelona, Spain (Reference No. PR/AG 223-2016). This trial was registered at www.clinicaltrials.gov under identification number NCT05128292. After receiving a verbal description of the study, all participants who enrolled in the study gave their signed informed written consent before initiating the study (before its commencement). This study was carried out in compliance with the 1975 Declaration of Helsinki, and all the International Conferences on Harmonization and Good Clinical Practice Guidelines.

### Participant's enrollment and eligibility criteria

Participants and patient-self-reported outcome measures were essentially as described previously (8). In brief, an 8-week prospective, open-label, single-arm exploratory clinical trial (CoSeME study) was conducted among 42 adult female ME/CFS patients consecutively enrolled at a single outpatient tertiary referral center (ME/CFS Clinical Unit, Vall d'Hebron University Hospital, Barcelona, Spain) from January through September 2018. All subjects were Caucasian, had a sedentary lifestyle, and were from the same geographical area at the time of the study. The overall study plan regarding patient screening and recruitment, along with experimental workflow before analysis, is displayed in Figure 1.

Demographic and clinical characteristics of the study participants are displayed in Table 1. Patients were potentially eligible if they were female, aged ≥18 years, and had a confirmed diagnosis of ME/CFS by a specialist according to the 1994 CDC/Fukuda criteria (9).

Exclusion criteria were those with any fatiguing-associated medical condition (thyroid-related disorders, sleep apnea, narcolepsy, medication side-effects, heart diseases, iron deficiency anemia), previous diagnosis not unequivocally resolved (chronic hepatitis, malignancy), autoimmune disorders, history of past/current neuropsychiatric disorders (major depressive disorder, psychotic or melancholic features, bipolar disorder, schizophrenia, delusional disorder, dementias, anorexia nervosa, and bulimia nervosa), and participation in another clinical trial of the same or different nature within 30 days before study inclusion; inability (in the opinion of the investigator) to follow the instructions or to complete the treatment satisfactorily; failure to provide signed informed consent; use of certain drugs and supplements that might influence outcome measures in the last 90 days or whose withdrawal might be a relevant problem, anticoagulant treatment, pregnancy or breast-feeding, smoking habits, alcohol intake or substance abuse, strong hormone-related medications, and obesity.

Participants with missing data from the final visit to baseline were considered to have dropped out.

### Product tested

Eligible patients meeting the inclusion/exclusion criteria were then instructed to take supplementation of 400 mg/day of CoQ10 soft-gel capsules (two Bio-Quinone Active^®^ 100 mg b.i.d.; Pharma Nord, Vejle, Denmark) distributed 30 min just before breakfast (two capsules) and lunch (two capsules), and one 200 μg/day of organic selenium yeast tablet (SelenoPrecise^®^ 200 μg; Pharma Nord) 30 min before breakfast over 8 weeks, after which the intervention was ended. The given supplementation was taken in addition to usual care and medication, if used. All study supplement boxes not consumed were returned and counted by an investigator (J.A-M.).

### Intervention procedures

Of the 42 eligible ME/CFS participants screened, 7 cases were excluded (4 who did not fulfill the inclusion criteria and 3 refused to participate). The remaining 35 participants were allocated to treatment by an independent investigator not otherwise involved in the intervention, using the result of a list of random numbers generated by a computer program. Height, weight, pulse, BP, and routine blood tests were taken at baseline and after 8 weeks of intervention from the participants.

Thirty-five subjects were consecutively assigned in a single-blind manner to receive either 200 mg of CoQ10 (ubiquinone) containing excipients (soybean oil, gelatine, glycerol [E422], purified water, d-alpha-tocopherol 1300 IU/g, and iron oxide [E172]) in soft-gel capsules twice daily (30 min before breakfast and lunch) plus one tablet of 200 μg of selenium daily (half an hour before breakfast) for 8 weeks (Fig. 1). The study pharmacist recorded all treatments supplied on the medication-dispensing forms along with the original script. The CoQ10 and selenium supplements were provided by Pharma Nord ApS.

Enrolled participants followed usual clinical care, in which they continued taking their standard medications as prescribed, with no medication changes mandated. Safety information obtained from baseline through the study end visit included data on adverse events, laboratory parameters, vital signs, along with the results of the general physical examination. Adverse events were to be reviewed throughout the intervention by the independent medical monitor, the steering committee, and the independent data and safety monitoring board. Provision was made for investigator-initiated temporary or permanent dose reductions or suspensions due to adverse effects. No adverse effects were noted.

### Outcomes

Participants were also asked to provide complete validated self-report questionnaires regarding their current health status at baseline and at 8-week follow-up intervention. Changes in symptom perception (fatigue, sleep abnormalities, and health-related quality of life) were assessed through self-reported symptom questionnaires filled in by each participant under the supervision of two trained investigators (J.C-M. and J.A-M) who oversaw compliance as described previously (8).

#### Fatigue perception

Fatigue severity was assessed using the FIS-40. Higher scores indicate greater functional limitations in daily life activities due to fatigue.

#### Sleep disturbances

Sleep disturbances were measured through the self-administered 19-item PSQI questionnaire. It contains seven domains on sleep quality: subjective sleep quality, sleep latency, sleep duration, habitual sleep efficiency, sleep perturbations, use of sleeping medication, and daytime dysfunction. The overall PSQI score ranges from 0 to 21 points, with scores of ≥5 indicating poorer sleep quality.

#### Short-form 36-item health survey

The SF-36 questionnaire was used to assess health-related quality of life. The SF-36 assesses functioning on eight components, including domains of physical functioning, physical role functioning, bodily pain, general health perception, vitality, social role functioning, emotional role functioning, and mental health. Lower scores indicate a more negative impact of an individual's health on functioning.

#### Collection and processing of blood samples

After an overnight fast, 10 mL peripheral blood was collected from an antecubital vein by venipuncture between 8:00 and 10:00 a.m. into 10 mL K_2_EDTA-containing plastic tubes (K_2_E Vacutainer; BD) after a resting time of 15 min in sitting position from each participant at baseline and after 8-weeks of intervention. Plasma was immediately separated by centrifugation at 2500 rpm for 15 min at 4°C (Beckman Coulter Centrifuge, MA), and stored in aliquots at −80°C until further laboratory analyses.

#### Laboratory analysis

Routine blood tests were measured by standard methods in the local core laboratory (Vall d'Hebron University Hospital, Clinical Biochemistry Lab, Barcelona, Spain). No sample was thawed more than twice. Repeated samples of each participant were measured in the same analytical batch.

### Biochemical measurements

#### Oxidative stress status

Assessment of oxidative damage was measured by TAC, which was expressed as μ*M* copper-reducing equivalents (Cat. No. STA-360) and lipid peroxidation (thiobarbituric acid-reacting substances or TBARS) assessed as MDA content (Cat. No. STA-330), which were assayed in plasma samples using the OxiSelect™ assay kit (Cell Biolabs, San Diego, CA) following the manufacturer's instructions.

#### Inflammatory cytokines assays

For simultaneous analysis of plasma cytokine levels (IL-1β, IL-6, IL-8, IL-10, TNF-α), a fluorescently labeled microsphere-based multiplex immunoassay was used and read on the Luminex-100 ISv2 system (Cat. No. HCYTOMAG-60K-05, Milliplex Map Human Cytokines/Chemokines; Linco Research/Millipore, Saint-Charles, MO). The intra- and interassay coefficients of variation for each cytokine were as follows: IL-1β: 7% and 12%; IL-6: 2% and 10%; IL-8: 3% and 14%; IL-10: 2% and 11%; and TNF-α: 3% and 19%, respectively.

#### CRP concentration

Plasma CRP concentration was determined by an immunoturbidometric assay using CRP Latex on a Beckman Coulter AU5800 automatic analyzer (Cat. No. OSR6199; Beckman Coulter, Brea, CA). The coefficient of variation was <5% for the range of CRP values reported in this study. Levels <0.5 mg/dL are considered normal values in healthy adults.

#### Quantification of FGF21 and NT-proBNP

Plasma FGF21 concentration was assayed using noncross-reactive enzyme-linked immunosorbent assays specific for the corresponding human protein (Cat. No. RD191108200R; BioVendor, Brno, Czech Republic). Levels of NT-proBNP were measured in plasma using a commercially available chemiluminescent immunoassay (Cat. No. 11200588, Atellica IM; Siemens Healthineers). Recommended clinical thresholds were 125 pg/mL for patients aged <75 years and 450 pg/mL for patients aged ≥75 years.

### Statistical analysis

Statistical analyses were carried out using the standard software (SPSS for Windows, v20.1; SPSS, Inc., Chicago, IL) in accordance with the appropriate tests for each variable considered. Power calculations, performed *a priori* based on our pilot data, indicated that ∼25 subjects would be required to detect at least a 20% change in perceived fatigue from baseline with 80% power using a one-tailed *t*-test at the 0.05 level of significance. Normality was confirmed using the Shapiro–Wilk test. The Kolmogorov–Smirnov and the Shapiro–Wilk tests were applied for the compatibility estimation of the studied parameters' distribution with normal distribution.

Nonparametric data were expressed as mean ± standard error of the mean (SEM) as shown in Table 1. Bar graphs show mean ± SEM including 25th and 75th percentiles of duplicate sampling. Comparisons between groups (baseline *vs.* post-treatment) were analyzed using the nonparametric Wilcoxon signed-rank test. Paired data with missing values were excluded from analysis. Two-sided *p* values <0.05 were considered statistically significant.

## Abbreviations Used

ALP: alkaline phosphatase
ALT: alanine aminotransferase
AST: aspartate aminotransferase
ATP: adenosine triphosphate
BMI: body mass index
BP: blood pressure
CCC: Canadian consensus criteria
CDC: Centers for Disease Control and Prevention
CoQ10: coenzyme Q10
CRP: C-reactive protein
FGF21: fibroblast growth factor 21
FIS-40: 40-item fatigue impact scale
GGT: gamma-glutamyl transferase
HDL: high-density lipoprotein
HR: heart rate
hs-CRP: high-sensitivity C-reactive protein
ICC: International Consensus Criteria for ME
IL: interleukin
IOM: Institute of Medicine from USA
LDL: low-density lipoprotein
MDA: malondialdehyde
ME/CFS: myalgic encephalomyelitis/chronic fatigue syndrome
NADH: nicotinamide adenine dinucleotide reduced
NIH: National Institute of Health
NSAID: nonsteroidal anti-inflammatory drug
NT-proBNP: N-terminal prohormone of brain natriuretic peptide
PSQI: Pittsburgh Sleep Quality Index
RBC: red blood cells
RCT: randomized-controlled trial
SEID: systemic exertion intolerance disease
SEM: standard error of the mean
SF-36: short-form 36-item health survey
T4: thyroxine
TAC: total antioxidant capacity
TBARS: thiobarbituric acid-reacting substances
TNF-α: tumor necrosis factor-alpha
TSH: thyroid-stimulating hormone
